# Antibiotic susceptibility and pathogenicity of *Aeromonas hydrophila* isolated from red hybrid tilapia (*Oreochromis niloticus*×*Oreochromis mossambicus*) in Malaysia

**DOI:** 10.14202/vetworld.2020.2166-2171

**Published:** 2020-10-16

**Authors:** Nurfarahin Ahmad Pauzi, Nurliyana Mohamad, Mohamad Azzam-Sayuti, Ina Salwany Md. Yasin, Mohd Zamri Saad, Nurrul Shaqinah Nasruddin, Mohammad Noor Amal Azmai

**Affiliations:** 1 Department of Biology, Faculty of Science, Universiti Putra Malaysia, 43400 UPM Serdang, Selangor, Malaysia; 2 Laboratory of Marine Biotechnology, Institute of Bioscience, Universiti Putra Malaysia, 43400 UPM Serdang, Selangor, Malaysia; 3 Department of Aquaculture, Faculty of Agriculture, Universiti Putra Malaysia, 43400 UPM Serdang, Selangor, Malaysia; 4 Department of Veterinary Laboratory Diagnosis, Faculty of Veterinary Medicine, Universiti Putra Malaysia, 43400 UPM Serdang, Selangor, Malaysia; 5 Department of Clinical Oral Biology, Faculty of Dentistry, Universiti Kebangsaan Malaysia, Jalan Raja Muda Abdul Aziz, 50300 Kuala Lumpur, Malaysia

**Keywords:** *Aeromonas hydrophila*, antibiotic sensitivity, aquaculture, pathogenicity, tilapia

## Abstract

**Background and Aim:**

*Aeromonas hydrophila* is a major cause of bacterial infections affecting a wide range of warm water fishes worldwide. In Malaysia, *A. hydrophila* isolations from diseased fishes were previously reported; however, with limited information. The present study investigates the antibiotic susceptibility and pathogenicity of *A. hydrophila* isolated from farmed red hybrid tilapia (*Oreochromis* spp.) in Malaysia.

**Materials and Methods:**

*A. hydrophila* was biochemically identified and subjected to antibiotic susceptibility tests. The isolate was then intraperitoneally injected into red hybrid tilapia, and the mortality, clinicopathological changes, and LD_50_ were determined up to 240 h post-infection (hpi).

**Results:**

The isolate demonstrated multiple antibiotic resistances (MAR) toward amikacin, ampicillin, cefotaxime, amoxicillin, trimethoprim-sulfamethoxazole, erythromycin, and streptomycin, with a MAR index of 0.5. The experimental infection of *A. hydrophila* at 10^5^ CFU/mL in the red hybrid tilapia resulted in 100% mortality at 240 hpi. The LD_50_ was determined at 1.1×10^4^ CFU/mL. Infected fish demonstrated occasional erratic swimming patterns, localized hemorrhages and depigmentation on the body and operculum areas, fin erosion, enlargement of the gall bladder, and hemorrhage in internal organs. Microscopic observation of infected fish revealed brain congestion, tubular necrosis, and glomerular shrinkage in the kidneys, necrosis of hepatocytes, and congestion of blood vessels in the liver.

**Conclusion:**

The high virulence of *A. hydrophila* to the red hybrid tilapia emphasizes the importance of active, on-going monitoring of its prevalence in Malaysian tilapia farming.

## Introduction

Tilapia (*Oreochromis* spp.) is among the major commercially important freshwater fish intensively farmed worldwide. World tilapia production has exceeded 5 million tonnes, generating incomes of approximately USD 9.8 billion in 2015, and has been increasing annually [1]. In Malaysia, the production of tilapia in 2017 was approximately 31,400 tonnes with a wholesale value of RM 286 million, which indicates the significance of tilapia farming in the country [2]. Although tilapia culture is steadily growing, the sector frequently suffers from disease outbreaks that represent a major obstacle to its growth.

*Aeromonas hydrophila* is recognized as a significant pathogen which can have a devastating impact on the fish farming industry [3]; it can be potentially ubiquitous in both marine and freshwater environments, and under favorable conditions, it has emerged as an opportunistic pathogen. Infection by *A. hydrophila* is usually characterized by hemorrhagic ulcers on the skin and fin erosion [3]. It has been previously reported in various freshwater fish including channel catfish (*Ictalurus punctatus*), Siberian sturgeon (*Acipenser baerii*), Russian sturgeon (*Acipenser gueldenstaedtii*), banded knifefish (*Gymnotus omarorum*), and Nile tilapia (*Oreochromis niloticus*) [4-6].

In Malaysia, occurrences of *A. hydrophila* have been frequently reported in aquatic environments and retail fish [7-9]. Moreover, the infection of red hybrid tilapia (*O. niloticus*×*Oreochromis mossambicus*) and catfish (*Clarias gariepinus*) by *A. hydrophila* has also been previously described [10,11]. However, the pathogenicity of the Malaysian *A. hydrophila* isolate has never been investigated.

In this study, we determined the antibiotic susceptibility and pathogenicity of *A. hydrophila* isolated from red hybrid tilapia in Malaysia.

## Materials and Methods

### Ethical approval

The handling of fish and all experimental procedures, in this study, was performed in accordance with the methods approved by the Institutional Animal Care and Use Committee, Universiti Putra Malaysia (AUP No.: R006/2016).

### Study period and location

The study was carried out for a period of 3 months from March until June 2019 at the Department of Biology, Faculty of Science, Universiti Putra Malaysia, Selangor, Malaysia.

### Bacterial strain

The *A. hydrophila* isolate used in this study was obtained from the Aquatic Laboratory, Institute of Bioscience, Universiti Putra Malaysia, Malaysia. The isolate had been previously isolated from the spleen of diseased red hybrid tilapia and identified as *A. hydrophila* using biochemical and molecular methods [12]. Glycerol stock (−80°C) of *A. hydrophila* was streaked on Tryptic Soy Agar (TSA) (Merck, Darmstadt, Germany) and incubated at 30°C for 24 h. A pure colony of the isolate was subjected to Gram staining, catalase testing, oxidase testing, and the use of an API 20NE test kit (bioMérieux, Marcy l’Etoile, France) for species confirmation.

Following the positive confirmation of *A. hydrophila*, the isolate was then intraperitoneally (IP) injected into three red hybrid tilapias (1 mL at 10^5^ CFU/mL of *A. hydrophila* per fish) for re-virulent purposes. The bacteria were re-isolated from moribund fish, cultured onto TSA, incubated at 30°C for 24 h, and reconfirmed as *A. hydrophila*.

### Antibiotic susceptibility testing

The *A. hydrophila* isolate was also subjected to antibiotic susceptibility tests, according to the guidelines proposed by the Clinical and Laboratory Standards Institute (CLSI) [13]. The commercial antibiotics used were amikacin (30 μg), ampicillin (10 μg), amoxicillin (30 μg), levofloxacin (5 μg), norfloxacin (10 μg), cefotaxime (30 μg), gentamicin (10 μg), kanamycin (30 μg), streptomycin (30 μg), erythromycin (15 μg), trimethoprim-sulfamethoxazole (1.25/23.75 μg), chloramphenicol (30 μg), tetracycline (30 μg), and nalidixic acid (30 μg) (Oxoid, London, UK). First, a fresh culture of *A. hydrophila* with a turbidity of 0.5 McFarland was swabbed onto the surface of Mueller-Hinton agar (MHA) (HiMedia, Mumbai, India) using sterile cotton buds. The antibiotic disks were fixed on the MHA surface using sterile forceps, and the agar plates were incubated at 35°C for 24 h. The inhibitory zones were interpreted according to the measurements provided by the CLSI guidelines [14]. The multiple antibiotic resistance (MAR) index was determined [15], and a MAR index value of >0.2 suggested a high-risk exposure to these antibiotics.

### Experimental infection study

For the pathogenicity study, ten colonies of 24 h cultures of *A. hydrophila* were inoculated into 100 mL of Tryptic Soy Broth (TSB) (Merck) and incubated at 30°C for 24 h. A ten-fold serial dilution was made with sterile TSB, and the standard spread-plate technique was used for bacterial enumeration.

One hundred eighty red hybrid tilapia (length: 9±2 cm) were purchased from local suppliers and kept in a tank (1500 L) for 1 week for acclimatization. No sign of disease or mortality was observed during the acclimatization period. Five fish were randomly sampled, and their gills and body surfaces were examined microscopically for the presence of parasites. Swabs were taken from internal organs and tested for bacterial presence. The samples were determined to be free from parasite and bacterial infection. Before a challenge test, the fish were distributed into five treatment groups and one control group of 10 fish. The experiment was conducted in triplicate. The fish in each of the five treatment groups were IP injected with 1 mL of *A. hydrophila* at concentrations increasing from 10^1^ to 10^5^ CFU/mL. The fish in the control group were injected with sterile TSB. Within 240 h post-infection (hpi), the clinical signs of gross lesions and mortality patterns were recorded, and the LD_50_ value was calculated [16]. Swabs were taken from the organs of the diseased red hybrid tilapia for isolation and identification of bacteria.

### Histopathological analysis

The livers, brains, kidneys, and eyes of moribund fish were collected and preserved in 10% buffered formalin for histopathological analysis. Briefly, the organs were processed using a tissue processor (Leica TP 1020, Leica, Germany), embedded in paraffin, sectioned at 4 μm thick (Leica Jung Multicut 2045, Germany), and stained with Harris’s hematoxylin and eosin. The sections were then examined under a light microscope (Nikon Eclipse 50i, Japan) and analyzed using Nikon NIS-Element D 3.2 Image Analysis software (Nikon Instruments Inc., USA).

## Results

Following identification using API 20NE, the result showed 99.9% similarity with *A. hydrophila*. The *A. hydrophila* isolate in this study formed yellowish, opaque colonies on TSA agar, produced beta-hemolysis on horse blood agar, and were motile, oxidase- and catalase-positive, and Gram-negative short rods (Table-1). The isolate tested positive for arginine dihydrolase and β-galactosidase, production of nitrate and indole, and hydrolysis of β-glucosidase and gelatin. It also assimilated arabinose, capric acid, glucose, malate, maltose, mannitol, mannose, N-acetylglucosamine, and potassium gluconate.

**Table 1:** Phenotypic and biochemical characteristics of *A. hydrophila* used in present study and its comparison with previously published *A. hydrophila* isolate.

Test	Phenotypic and biochemical characteristics	

	*A. hydrophila* (present study)	*A. hydrophila* [17]
Gram stain	− (Short rod)	− (Short rod)
Hemolysis (horse blood)	Beta-hemolysis	Beta-hemolysis
Oxidase	+	+
Catalase	+	+
Motility	+	+
Reduction of nitrates to nitrites	+	+
Indole production	+	+
Fermentation of glucose*	−	+
Arginine dihydrolase	+	+
Urease	−	NA
Hydrolysis of β-glucosidase	+	+
Hydrolysis of protease (gelatin)	+	+
β-galactosidase	+	+
Assimilation of adipic acid*	−	+
Assimilation of arabinose	+	+
Assimilation of capric acid	+	+
Assimilation of glucose	+	+
Assimilation of malate	+	+
Assimilation of maltose	+	+
Assimilation of mannitol	+	+
Assimilation of mannose	+	+
Assimilation of (N-acetyl-Glucosamine)	+	+
Assimilation of phenylacetic acid	−	−
Assimilation of potassium gluconate	+	+
Assimilation of trisodium citrate	−	−

+=Positive, −=Negative, NA=Not available. All biochemical tests are included in the API 20NE.

* Indicate difference of characteristics from previous study. *A. hydrophila=Aeromonas hydrophila*

The *A. hydrophila* isolate was sensitive to levofloxacin (inhibition zone: 24 mm), gentamicin (18 mm), chloramphenicol (23 mm), tetracycline (16 mm), nalidixic acid (23 mm), kanamycin (18 mm), and norfloxacin (22 mm) and resistant to amikacin (0 mm), ampicillin (0 mm), cefotaxime (14 mm), amoxicillin (0 mm), trimethoprim-sulfamethoxazole (0 mm), erythromycin (0 mm), and streptomycin (0 mm).

**Figure-1:**
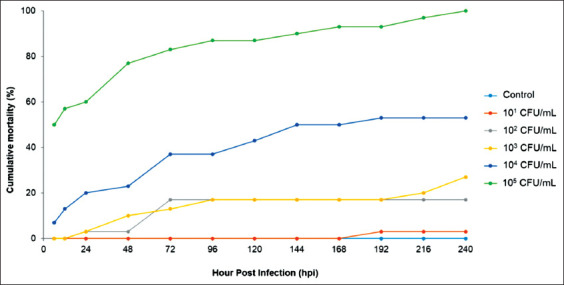
Mortality patterns of experimentally infected red hybrid tilapia with different concentration of *Aeromonas hydrophila*. No mortality was observed in control group injected with sterile Tryptic Soy Broth.

In the pathogenicity study, the earliest mortality was observed at 6 hpi in the groups infected with 10^4^ CFU/mL and 10^5^ CFU/mL of *A. hydrophila* (Figure-1). The highest cumulative mortality (100%) was observed in the group infected with 10^5^ CFU/mL, followed by group infected with 10^4^ CFU/mL (53.3%), 10^3^ CFU/mL (26.7%), 10^2^ CFU/mL (16.7%), and 10^1^ CFU/mL (3.3%). In the present study, the calculated LD_50_ of *A*. *hydrophila* was 1.1×10^4^ CFU/mL.

**Figure-2:**
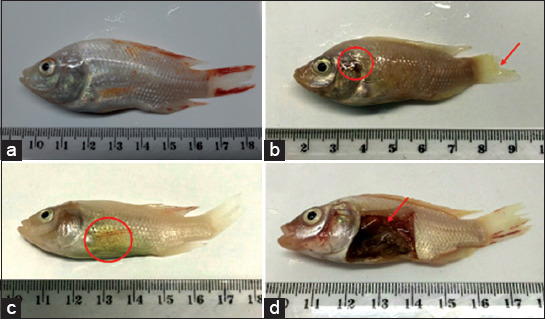
Clinical signs of red hybrid tilapia following *Aeromonas hydrophila* infection. (a) Healthy control fish showing no clinical signs and abnormalities; (b) hemorrhages around the operculum area (circle) and ulceration at caudal fin (arrow); (c) enlarged gall bladder; (d) intra-peritoneal hemorrhages.

Following IP injection, infected tilapia exhibited occasional erratic swimming patterns; hemorrhagic foci and depigmentation on body and operculum areas and tail erosion were also observed (Figure-2). Internally, infected fish demonstrated enlarged gall bladders and hemorrhage of internal organs. *A. hydrophila* was successfully isolated from the freshly dead fish.

**Figure-3:**
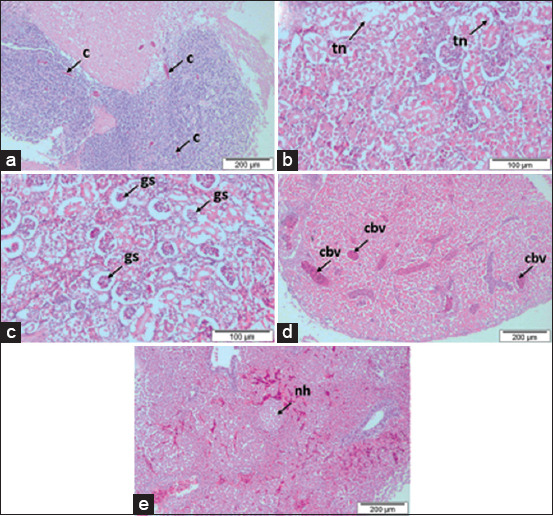
Histopathological changes of red hybrid tilapia infected by *Aeromonas hydrophila*. (a) Mild-moderate congestion (c) in brain; (b) mild-moderate tubular necrosis (tn) in the kidney; (c) mild observation of glomerular shrinkage (gs) in the kidney; (d) mild-moderate congestion of blood vessels (cbv) in the liver; (e) mild-moderate necrosis of the hepatocytes (nh) in the liver.

Microscopic observation of infected red hybrid tilapia brain tissue revealed mild-to-moderate congestion (Figure-3). In addition, mild-to-moderate tubular necrosis and glomerular shrinkage in the kidneys and mild-to-moderate necrosis of hepatocytes and congestion of blood vessels in the livers of infected red hybrid tilapia were observed.

## Discussion

This study reports the antibiotic susceptibility and pathogenicity of *A. hydrophila* isolated from diseased red hybrid tilapia in Malaysia. *A. hydrophila* has been responsible for significant losses in the tilapia aquaculture industry worldwide. In Malaysia, its presence has been reported in the aquatic environment, retail, and cultured fish [7-10]; however, the pathogenicity of local *A. hydrophila* isolate has never been investigated.

The morphological and biochemical characteristics of *A. hydrophila* observed in this study are consistent with previous reports [17], except for its inability to ferment glucose and assimilate adipic acid. The isolate demonstrated multiple resistances toward the tested antibiotics including amikacin, ampicillin, cefotaxime, amoxicillin, trimethoprim-sulfamethoxazole, erythromycin, and streptomycin with a MAR index of 0.5. The resistance of *A. hydrophila* toward the penicillin group has been previously reported in Thailand [18], India [19], and Pakistan [17], probably due to the prolonged and excessive use of these antibiotics. Significantly, in this study, the quinolone group of antibiotics successfully inhibited the growth of *A. hydrophila*; hence, it can be utilized as a treatment in tilapia farms. However, with the concerns of newly emerging MAR pathogens, the responsible use of antibiotics in fish farming should be adopted, and alternative treatments to combat *A. hydrophila* infection should be applied.

The pathogenicity study revealed that red hybrid tilapia was susceptible to *A. hydrophila* infection following IP injection. The previous experimental studies on *A. hydrophila* infection in various hosts showed cumulative mortality ranging from 60% to 100%, depending on the challenge doses and route of infection [20-23], which indicates a wide range of fish host susceptible to this pathogen. In this study, the LD_50_ was determined at 1.1×10^4^ CFU/mL, suggesting a low tolerance of red hybrid tilapia to *A. hydrophila* infection. In previous studies on the experimental infection of *A. hydrophila* using IP routes, the LD_50_ were recorded at 4.1×10^8^ CFU/mL for snakehead fish (*Channa striata*) [23] and 4.53×10^6^ CFU/mL for gourami (*Osphronemus goramy*) [22].

In the present study, the observed clinical signs were similar to those observed in affected catfish and Nile tilapia in natural outbreaks of *A. hydrophila* infection [10,24] but with the lower severity. Other gross lesions such as exophthalmia and the presence of ascites caused by *A. hydrophila* infection in fish were not observed in this study [10,24]. Highly pathogenic strains may cause fish to die quickly without showing apparent signs of disease, as observed in this study where mortality started early at 6 hpi. In addition, other factors, such as the type and age of the host and the time of exposure, can also influence the severity of the clinical symptoms [5,6]. This study revealed that the kidneys of infected red hybrid tilapia showed tubular necrosis and glomerular shrinkage that was similar to previous reports [10,25]. Necrosis of the hepatocytes and congestion of blood vessels in the livers of infected red hybrid tilapia have also been observed in infected golden mahseer (*Tor putitora*) [26] and channel catfish [25].

## Conclusion

This study demonstrates the susceptibility of red hybrid tilapia to *A. hydrophila* infection. Apart from its threat to cultured fish, the MAR of *A. hydrophila* isolates may also pose a health threat to humans. With respect to the potential pathogenicity of *A. hydrophila* to tilapia, more attention should be given to the diagnostics of the disease in farms and the effective control of this pathogen through the use of appropriate and environment-friendly therapeutic measures.

## Authors’ Contributions

NAP and MA conducted the experimental studies, analyzed and interpreted the data. NM, NSN, and MNAA contributed in the data interpretation. NM and MNAA drafted the manuscript. ISMY, MZS, NSN, and MNAA were involved in critical reading and editing. All authors read and approved the final manuscript.

## References

[1] Food and Agriculture Organization of the United Nations (2017). FishStatJ, a Tool for Fishery Statistics Analysis. FAO Fisheries and Aquaculture Department, FIPS Statistics and Information, Rome. http://www.fao.org/fishery/statistics/software/fishstatj/en..

[2] Annual Fisheries Statistic (2018). Annual Fisheries Statistic. Department of Fisheries Malaysia, Ministry of Agriculture and Agro-Based Industry, Malaysia. https://www.dof.gov.my.

[3] Harikrishnan R, Balasundaram C (2005). Modern trends in *Aeromonas hydrophila* disease management with fish. Rev. Fish. Sci.

[4] Aboyadak I.M, Ali N.G.M, Goda A.M.A, Aboelgalagel W.H, Salam A (2015). Molecular detection of *Aeromonas hydrophila* as the main cause of outbreak in tilapia farms in Egypt. J Aquac. Mar. Biol.

[5] Zhang D, Xu D.H, Shoemaker C (2016). Experimental induction of motile *Aeromonas* septicemia in channel catfish (*Ictalurus punctatus*) by waterborne challenge with virulent *Aeromonas hydrophila*. Aquac. Rep.

[6] Perretta A, Antúnez K, Zunino P (2018). Phenotypic, molecular and pathological characterization of motile aeromonads isolated from diseased fishes cultured in Uruguay. J. Fish Dis.

[7] Radu S, Ahmad N, Ling F.H, Reezal A (2003). Prevalence and resistance to antibiotics for *Aeromonas* species from retail fish in Malaysia. Int. J. Food Microbiol.

[8] Khor W.C, Puah S.M, Tan J.A.M, Puthucheary S.D, Chua K.H (2015). Phenotypic and genetic diversity of *Aeromonas* species isolated from freshwater lakes in Malaysia. PLoS One.

[9] Hamid R, Ahmad A, Usup G (2016). Pathogenicity of *Aeromonas hydrophila* isolated from the Malaysian Sea against coral (*Turbinaria* sp.) and sea bass (*Lates calcarifer*). Environ. Sci. Pollut. Res.

[10] Laith A.R, Najiah M (2013). *Aeromonas hydrophila*:Antimicrobial susceptibility and histopathology of isolates from diseased catfish. *Clarias gariepinus*(Burchell). J. Aquac. Res. Dev.

[11] Lee S.W, Wendy W (2017). Antibiotic and heavy metal resistance of *Aeromonas hydrophila* and *Edwardsiella tarda* isolated from red hybrid tilapia (*Oreochromis* spp.) coinfected with motile aeromonas septicemia and edwardsiellosis. Vet. World.

[12] Saleema M (2015). Molecular Characterization of *Aeromonas hydrophila* and Development of Recombinant Cells Vaccine Expressing outer Membrane Proteins against its in African Catfish (*Clarias gariepinus* Burchell) (Unpublished Master Thesis).

[13] Clinical and Laboratory Standards Institute (2016). Methods for Dilution Antimicrobial Dilution and Disk Susceptibility Testing of Infrequently Isolated or Fastidious Bacteria;Approved Guideline. https://www.clsi.org.

[14] Clinical and Laboratory Standards Institute (2013). Performance standards for Antimicrobial Susceptibility Testing;Twenty-third informational supplement. CLSI document M100-S23. Clinical and Laboratory Standards Institute, Wayne, Pennsylvania, USA. Available from:https://www.clsi.org. Retrieved on 03-07-2019.

[15] Krumperman P.H (1985). Multiple antibiotic indexing of *E. coli* to identify high-risk sources of fecal contamination of foods. Appl. Environ. Microbiol.

[16] Reed L.J, Muench H (1938). A simple method of estimating fifty percent endpoints. Am. J. Epidemiol.

[17] Ali S, Akhter S, Muhammad A, Khan I, Khan W.A, Iqbal M.N, Umar S, Ahmed H, Ali Q (2016). Identification, characterization and antibiotic sensitivity of *Aeromonas hydrophila* a causative agent of epizootic ulcerative syndrome in wild and farmed fish from Potohar, Pakistan. Pak. J. Zool.

[18] Tipmongkolsilp N, del Castillo C.S, Hikima J.I, Jung T.S, Kondo H, Hirono I, Aoki T (2012). Multiple drug-resistant strains of *Aeromonas hydrophila* isolated from tilapia farms in Thailand. Fish Pathol.

[19] Samal S.K, Das B.K, Pal B.B (2014). Isolation, biochemical characterization, antibiotic susceptibility study of *Aeromonas hydrophila* isolated from freshwater fish. Int. J. Curr. Microbiol. Appl. Sci.

[20] Hossain M.F, Rashid M.M, Sayed M.A (2011). Experimental infection of indigenous climbing perch (*Anabas testudineus*) with *Aeromonas hydrophila* bacteria. Prog. Agric.

[21] Dias M.K.R, Sampaio L.S, Proietti-Junior A.A, Yoshioka E.T.O, Rodrigues D.P, Rodriguez A.F.R, Tavares-Dias M (2016). Lethal dose and clinical signs of *Aeromonas hydrophila* in *Arapaima gigas*(*Arapaimidae*), the giant fish from Amazon. Vet. Microbiol.

[22] Rozi Rahayu, K Daruti D.N, Stella M.S.P (2018). Study on characterization, pathogenicity and histopathology of disease caused by *Aeromonas hydrophila* in gourami (*Osphronemus gouramy*). Earth Environ. Sci.

[23] Samayanpaulraj V, Velu V, Uthandakalaipandiyan R (2019). Determination of lethal dose of *Aeromonas hydrophila* Ah17 strain in snake head fish *Channa striata*. Microb. Pathog.

[24] El Deen A.N, Dorgham-Sohad M, Hassan-Azza H.M, Hakim A.S (2014). Studies on *Aeromonas hydrophila* in cultured *Oreochromis niloticus* at Kafr El Sheikh Governorate, Egypt with reference to histopathological alterations in some vital organs. World J. Fish Mar. Sci.

[25] Abdelhamed H, Ibrahim I, Baumgartner W, Lawrence M.L, Karsi A (2017). Characterization of histopathological and ultrastructural changes in channel catfish experimentally infected with virulent *Aeromonas hydrophila*. Front. Microbiol.

[26] Kumar R, Pande V, Singh L, Sharma L, Saxena N, Thakuria D, Singh A.K, Sahoo P.K (2016). Pathological findings of experimental *Aeromonas hydrophila* infection in golden mahseer (*Tor putitora*). Fish. Aquac. J.

